# Editorial: Use of Barley and Wheat Reference Sequences: Downstream Applications in Breeding, Gene Isolation, GWAS, and Evolution

**DOI:** 10.3389/fpls.2020.01017

**Published:** 2020-07-07

**Authors:** Dragan Perovic, Hikmet Budak, Kazuhiro Sato, Pierre Sourdille

**Affiliations:** ^1^ Federal Research Centre for Cultivated Plants, Institute for Resistance Research and Stress Tolerance, Julius Kuehn Institute, Quedlinburg, Germany; ^2^ Montana BioAgriculture, Inc., Bozeman, MT, United States; ^3^ Institute of Plant Science and Resources, Okayama University, Kurashiki, Japan; ^4^ INRAE, UMR 1095 INRAE—UCA Genetics, Diversity & Ecophysiology of Cereals, Clermont-Ferrand, France

**Keywords:** barley, breeding, gene isolation, genome reference sequence, wheat

Barley and wheat are the most important temperate cereal crops of the Triticeae. Although they ranked in terms of world production, fourth [barley 141 million tons (MT)] and second (wheat 749 MT), according to FAO (2018), their large genomes prevented them from being fully sequenced until recently. Nevertheless, advances in the development of new high-throughput sequencing technologies together with efforts of scientific communities enabled accomplishment of this long-standing aims (Mascher et al., 2017; IWGSC, 2018). Nowadays, the availability of these high-standard reference genomes has ushered in a new era of barley and wheat genomics, and their decoded blueprints open new avenues in exploring genome sequences for both applied and basic research. Barley and wheat geneticists and breeders are presently in a position in which users of the first sequenced model plants, rice, and Arabidopsis, were nearly twenty years ago. The current Frontiers in Plant Science research collection of 18 articles sheds light on how knowledge of whole barley and wheat genome sequences promotes applied breeding [Genome Wide Association Study (GWAS), genomic selection (GS)], basic biological research [mapping of major genes and quantitative trait loci (QTLs)], accelerated isolation of novel genes, novel methods in sequence analysis, and rapid detection of natural variation. Furthermore, the presented articles revealed efficient use of genetics and breeding methods in harnessing genetic resources of barley and wheat that promote the rapid improvement of cultivars.

Ladejobi et al. demonstrated that using this resource for alignment of Genotyping-by-Sequencing (GBS) reads and variant Single Nucleotide Polymorphism (SNP) calling enabled the generation of thousands of high-quality SNP data points. When applied to association mapping and genomic prediction, GBS data anchored to wheat IWGSC RefSeq v1.0 generally improved prediction accuracy. In particular, this study demonstrated the utility of GBS reads for efficiently predicting traits with numerous loci each having a small effect, proving its suitability for GS.

Furthermore, Alomari et al. showed the power of the GWAS approach for identifying putative candidate genes for Zn accumulation into the grain using 369 European wheat varieties genotyped with high-density arrays of SNP markers (90k iSelect Infinium and 35k Affymetrix arrays) in combination with the wheat IWGSC Ref Seq v1.0. This study discovered two physically anchored chromosomal segments located on chromosomes 3B and 5A, as genetic factors controlling Zn accumulation into the grain. These genomic regions include newly identified putative candidate genes, which are related to Zn uptake and transport or represent bZIP and mitogen-activated protein kinase genes.

Wang et al. applied GWAS in 493 durum worldwide collection to address the genetic basis of 17 agronomically important traits and a drought wilting score. Based on sequence alignment of the markers to the reference genome of bread wheat, they identified 14 putative candidate genes involved in enzymes, hormone-response, and transcription factors. The GWAS in durum wheat and a previous QTL analysis in bread wheat identified a consensus QTL locus.4B.1 conferring drought tolerance, which was further scanned for the presence of potential candidate genes.

Sheoran et al. used GWAS in a diverse panel of 404 spring wheat genotypes in India. By using Breeders’ 35K SNP Axiom array covering 4364.79 cM of the wheat genome, a total of 146 SNPs were found associated with 23 out of 36 studied traits explaining 3.7–47.0% of phenotypic variance. Gene annotation mined ∼38 putative candidate genes, which were confirmed using tissue and stage specific gene expression derived from RNA Seq data. They observed strong colocalized loci for four traits on chromosome 1B and annotated five putative candidate genes.

Fan et al. identified that QTLs for six spike-related traits under two different nitrogen (N) supplies, based on a high-density genetic linkage map constructed using PCR markers, DArTs, and Affymetrix Wheat 660 K SNP array. A total of 157 traditional QTLs and 54 conditional loci were detected by inclusive composite interval mapping, among which three completely low N-stress induced QTLs were found to maintain the desired spikelet fertility and kernel numbers even under N deficiency through pyramiding elite alleles.

Liu et al. conducted selection signal detection and GWAS for spike related traits in common wheat. Based on the genotyping results (90K SNP array), 192 common wheat samples from southwest China were analyzed. A total of 146 selective windows and 184 significant SNPs were detected. According to the wheat RefSeq v1.0, these SNP clusters and their overlapping/flanking QTLs that were previously reported were integrated to a physical map. According to the haplotype analysis, KASP markers were developed.

Martin et al. presented an extensive analysis of RNA-seq data in the presence and absence of the *Ph*1 locus in order to find out how this gene likely modified the meiotic process and plays a role in polyploidy adaptation. Plant material from an early prophase from six different genotypes (wheat, wheat–rye haploid hybrids and newly synthesized octoploid triticale) unexpectedly revealed that neither synapsis, whole genome duplication nor the absence of the *Ph*1 locus was associated with major changes in gene expression levels during early meiotic prophase. Overall, results of this study suggested that wheat transcription at this meiotic stage is highly resilient to such alterations, even in the presence of major chromatin structural changes.

Six articles describe characterization and use of germplasm, wild relatives’ introgressions, a-gene stocks.

Monteagudo et al. showed that Spanish barley landraces contribute to the improvement of elite cultivars as donors of novel alleles/genes of agronomically important traits such as flowering time, yield, and drought-related traits. In specific cases, they could become cultivars directly or at least could be used as parents in plant breeding programs due to their reduced genetic load.

Dinglasan et al. characterized and mapped resistance to net form of net blotch [*Pyrenophora teres f. teres* (Ptt)] in the international barley differential cv. Canadian Lake Shore (CLS) using a doubled haploid (DH) population. The authors identified a major QTL (qPttCLS) on chromosome 3H conferring resistance to Ptt, while aligning DArTseq markers to the barley physical-map position allowed identification of annotated genes

Dracatos et al. used GBS for the resistance to rust diseases in barley. They produced a high-density linkage map comprising 8,610 (SNPs and *in silico*) markers spanning 5957.6 cM to map resistance to leaf rust, stem rust, and stripe rust.

In wheat, Mia et al. reported fast track development and evaluation of Near Isogenic Lines (NILs) from C306 × Dharwar Dry targeting a wheat 4BS QTL hotspot in C306, which confers drought tolerance following the heterogeneous inbreed family (HIF) analysis coupled with immature embryo culture-based fast generation technique. Quantitative RT-PCR analysis targeting the MYB 82 transcription factor (TaMYB82), within this genomic region, also revealed differential expression in +NILs and −NILs under stress.

Synthetic wheats were also analyzed. Naz et al. evaluated two advanced backcross populations B22 and Z86, which were derived by crossing winter wheat cultivars Batis and Zentos with synthetic hexaploid wheat accessions Syn022L and Syn086L, respectively. QTL analysis identified seven and 13 favorable exotic QTL alleles associated with enhancement or at least stable grain yield in populations B22 and Z86, respectively. These favorable introgressions were located on all chromosomes from 1D to 7D.

Khalid et al. analyzed a diversity panel consisting in advanced lines derived from synthetic hexaploid wheats for allelic variation at 87 functional genes or loci of breeding importance using 124 high-throughput KASP markers. The major developmental genes such as *Vrn-A1*, *Rht-D1*, and *Ppd-B1* had confounding effect on several agronomic traits including plant height, grain size and weight, and grain yield in both well-watered (WW) and water-limited (WL) conditions.

Positional cloning of genes is among those activities that benefit most from an anchored and annotated genome sequence. Articles of Fazlikhani et al. and Hoseinzadeh et al. showed that the resistance gene isolation in barley might be faster from gene mapping to the identification and functional validation of candidate gene. In particular, the barley reference sequence delivered detailed information about the physical size of the target intervals, while the respective gene annotation (Mascher et al., 2017) revealed the genes located in the target intervals and putative candidate genes, as well as facilitated the efficient development of molecular markers for marker-assisted selection. Allele specific resequencing of putative candidate genes and construction of corresponding haplotypes are nowadays much accelerated and easier.

Three articles presented methods for the use of reference genome sequences for characterization of genetic resources, development of molecular markers, and identification of noncoding RNA structures.

Keilwagen et al. demonstrated that deep-coverage analysis of GBS data combined with mapping of reads on reference sequences results in the detection of *Hordeum vulgare*/*Hordeum bulbosum* introgression lines as well as to identify large chromosomal rearrangements in barley and wheat collections. In addition, the method is useful to identify genomic regions under selection and could be applied to control for duplicates in gene bank collections.

The availability of reference genome sequence facilitates the generation of markers by elucidating the genomic positions of new markers as well as of their neighboring sequences. Tanaka et al. showed that RNA-Seq-based *de novo* polymorphism detection system generates genome-wide markers, even in the closely related barley genotypes used in breeding programs.

In wheat and barley, the knowledge about lncRNAs remains very limited. Budak et al. showed that the high-quality reference genomes of wheat and barley significantly help to reduce false annotation of lncRNAs, and to obtain a well-assembled transcriptome data will greatly advance the lncRNA identification procedures. Therefore, high-quality genome sequences are promising resources for the identification of lncRNAs or any class of molecules. As our understanding of lncRNAs expands, interactions among ncRNA classes, as well as interactions with the coding sequences, will likely define novel functional networks that may be modulated for crop improvement.

In short, this topic integrated the different genomic approaches in combination with the biological and agronomic information of importance in two of the most important cereal crops, barley and wheat, in order to provide with new tools and methodologies that allow a great leap forward in plant breeding.

## Future Perspectives

The availability of genome sequences revolutionized barley and wheat genetics, accelerated identification and use of rare allelic variants in classical breeding schemes, such as marker-assisted backcrossing (MABC), marker-assisted selection (MAS) and pyramiding of genetic factors responsible for important traits. Furthermore, the availability of newly developed genomic tools and resources is leading to a new revolution of plant breeding, as they facilitate the study of the genotype and its relationship with the phenotype. After sequencing of more accessions, as a result of PanGenome projects, it will be possible to do direct targeting of important gene variants and introduce them into cultivars in order to exploit the rich germplasms for breeding purposes. In this regard, new breeding technologies such as site-directed mutagenesis by RNA-guided endonucleases like Cas9 bear possibilities. For example, alleles, identified by new genomic approaches, can be mimicked in breeding lines to circumvent time-consuming crosses.

## Author Contributions

DP prepared the first draft of this editorial. All authors contributed to the article and approved the submitted version.

## Conflict of Interest

Author HB was employed by company Montana BioAg. Inc.

The remaining authors declare that the research was conducted in the absence of any commercial or financial relationships that could be construed as a potential conflict of interest.
